# Biocompatibility Research of a Novel pH Sensitive Ion Exchange Resin Microsphere 

**Published:** 2014

**Authors:** Hongfei Liu, Shuangshuang Shi, Weisan Pan, Changshan Sun, Xiaomian Zou, Min Fu, Yingshu Feng, Hui Ding

**Affiliations:** a College of Pharmacy, Jiangsu University, Zhenjiang, 212013, China.; b Department of Pharmaceutics, Shen Yang Pharmaceutical University, Shen Yang 110016, China.; c Department of Pharmaceutical Analysis, China Pharmaceutical University, Nanjing,China.

**Keywords:** pH sensitive ion exchange resin, Pharmacology, Toxicology, Biocompatibility, Microsphere

## Abstract

The main objective of this study was to investigate biocompatibility and provide *in-vivo *pharmacological and toxicological evidence for further investigation of the possibility of pH sensitive ion exchange resin microsphere for clinical utilizations. Acute toxicity study and general pharmacological studies were conducted on the pH sensitive ion exchange resin microsphere we prepared. The general pharmacological studies consist of the effects of the pH sensitive ion exchange resin microsphere on the nervous system of mice, the functional coordination of mice, the hypnosis of mice treated with nembutal at subliminal dose, the autonomic activities of tested mice, and the heart rate, blood pressure, ECG and breathing of the anesthetic cats. The LD_50 _of pH sensitive ion exchange resin microsphere after oral administration was more than 18.84 g·Kg^-1^. Mice were orally administered with 16 mg·Kg^-1^, 32 mg·Kg^-1^ and 64 mg·Kg^-1^ of pH sensitive ion exchange resin microsphere and there was no significant influence on mice nervous system, general behavior, function coordination, hypnotic effect treated with nembutal at subliminal dose and frequency of autonomic activities. Within the 90 min after 5 mg·Kg^-1^, 10 mg·Kg^-1^, 20 mg·Kg^-1^ pH sensitive ion exchange resin microsphere was injected to cat duodenum, the heart rate, blood pressure, breathing and ECG of the cats didn’t make significant changes in each experimental group compared with the control group. The desirable pharmacological and toxicological behaviors of the pH sensitive ion exchange resin microsphere exhibited that it has safe biocompatibility and is possible for clinical use.

## Introduction

The main purpose of drug delivery research is to develop formulations that meet the therapeutic needs related to particular pathological conditions (1). Biological rhythms have to be taken into account when evaluating drug delivery systems, galenic formulations and pharmacokinetics as a basis for drug treatment (2). As is known, the symptomatology of some diseases exhibits circadian rhythms along with physiological functions. In particular, symptoms of asthma (3), arthritis (4) and epilepsy (5) appear to have a peak during the night or early in the morning. To improve patients’ compliance, pulsatile release formulations which can release drugs after a period of lag-time are developed. Traditional pulsatile release formulations are mainly tablets coated with a layer of semipermeable membrane to improve sustained release profiles. However, if the tablet is broken before it is taken, there is a risk that burst release would occur in patients, accompanied with serious side-effects. To overcome these shortcomings, polydispersity systems such as microcapsule, pellet and microsphere have recently been an attractive choice for developing sustained release systems. 

Ion exchange resins are high-molecular weight polyelectrolytes, which can exchange mobile ions of similar charge with the surrounding medium. Recently, they have been widely used as drug delivery carriers (6-8). The ionic interactions are strongly dependent on the pH and the competing ions in the reaction medium. If the medium has many ionic species, it may decrease the electrostatic interaction between the resin and the ionic drug due to shielding and competitive binding effect (7), which enables its potential for sustained release and pulsatile release. Furthermore, the modification of the structure of the ion exchange resins can offer additional advantages beyond the inherent improved properties of the ion exchange resin itself, including pH sensitive ion exchange resin in particular.

In our previous studies, we successfully prepared and characterized a novel pH sensitive ion exchange resin (9), and investigated its release profiles and pharmacokinetics using metformin hydrochloride and salbutamol sulfate as model drugs (10-11).

In this study, to further investigate the possibility of pH sensitive ion exchange resin for clinical utilizations, we carried out pharmacological and toxicological studies. Acute toxicity study and general pharmacological studies were conducted on the pH sensitive ion exchange resin we self-made. The general pharmacological studies consist of the effects of the pH sensitive ion exchange resin on the nervous system of mice, the functional coordination of mice, the hypnosis of mice treated with nembutal at subliminal dose, the autonomic activities of tested mice, and the heart rate, blood pressure, ECG and breathing of the anesthetic cats. Animals including mice and cats were used to finish these experiments. This study will provide valuable messages for the potential clinical applications of this novel pH sensitive ion exchange resin.

## Experimental

*Materials*

pH sensitive ion exchange resin microsphere was prepared in our lab. Nembutal was purchased from Shanghai Chemical Reagent Company of China Pharmaceutical Group (Shanghai, China, batch number: F20030816). Animals were kindly provided by the Experimental Animal Center of Shenyang Pharmaceutical University (Liaoning, China), including male Kunming rats weighing 250 ± 20 g, male New Zealand white rabbits weighing 2.1 Kg (license: SYXK (Liaoning) 2011-0013), male and female guinea pigs weighing 300~350 g (license: SYXK (Liaoning) 2011-0013), and male and female Kunming mice weighing 18-22 g (license: SCXK (Liaoning) 2011-009). Cats were purchased from the market by the Experimental Animal Center of Shenyang Pharmaceutical University. Autonomic activity tester for mice was provided by Beijing pharmaceutical institute (ZIR-2, Beijing, China). RM6240CD multi-channel bio-signal acquisition and processing system was purchased from Chengdu Instrument Factory (Sichuan, China).

*Acute toxicity test by oral administration (* 12 *)*

After fasted but available of water for 16 hours, 40 mice were randomly divided into two groups: the experimental group and the control group, with 20 mice in each group. With the maximum concentration (0.471 g·mL^-1^) and maximum allowable volume (0.8 mL/20 g), pH sensitive ion exchange resin (0.5% CMC-Na solution as medium) was administered to the experimental group, while the control group was given the same volume of 0.5% CMC-Na solution. After 8 hours, the administration was repeated once with the same concentration and volume. Then the mice were observed for 2 weeks. 

*General pharmacological studies (* 13 *)*

*The effect of pH sensitive ion exchange resin on nervous system of mice after oral administration*

The tested mice were divided into four groups at random, five male and five female mice for each group. The pH sensitive ion exchange resin with 16 mg·Kg^-1^, 32 mg·Kg^-1^, 64 mg·Kg^-1^ (0.5% CMC-Na as the medium) was orally administered to the three experimental groups with the volume of 0.1 mL/ 10 g. The control group was orally administered with the same volume of 0.5% CMC-Na. Irwin behavior experiment was employed to investigate the influence of pH sensitive ion exchange resin on the behavior of mice including righting reflex, passive state, muscle twitching, salivation and nystagmus. The mice were observed before administration, 5, 10, 30, 60 and 90 min after administration. 

*The effect of pH sensitive ion exchange resin on the functional coordination of mice (rotating rods method)*

The tested mice were screened. The mice which can climb up for 3 min on rotating rods were qualified. The qualified mice were then divided into four groups at random, half male and half female in each group. The pH sensitive ion exchange resin with 16 mg·Kg^-1^, 32 mg·Kg^-1^, 64 mg·Kg^-1^ (0.5% CMC-Na as the medium) was orally administered to the three experimental groups with the volume of 0.1 mL/10 g. The control group was orally administered with the same volume of 0.5% CMC-Na. At 30 min after administration, mice were put on rotating rods (16 r·min^-1^) to calculate the percentage of mice which fell within 1 min. 

*The effect of pH sensitive ion exchange resin on hypnosis of mice treated with nembutal at subliminal dose*

80 mice were divided into four groups evenly with the same condition of sex and weight, each group containing ten male and ten female. The control group was administered with 0.5% CMC-Na by oral administration, while the rest three groups were administered with pH sensitive ion exchange resin using the dose 16 mg·Kg^-1^, 32 mg·Kg^-1^ and 64 mg·Kg^-1^ respectively with 0.1 mL/ 10 g. After 30 min, 25 mg·Kg^-1^ nembutal was intraperitoneally injected to each tested mouse with 0.1 mL/ 10 g. If the righting reflex disappeared for 1 min, the mouse was regarded as being asleep. Each group was observed for the number of mice getting asleep at 30 min after the injection of nembutal. The experimental groups and control group were compared by X^2 ^testing.

*The effect of pH sensitive ion exchange resin on the autonomic activities of tested mice*

The tested mice were divided into four groups at random, five male and five female in each group. Before the determination of autonomic activity, the mice were put on the tester (Beijing drug research institute) for 3 min to be adapted. The mice were recorded for their frequency of autonomic activity within 3 min as the index before administration. Then each experimental group was given pH sensitive ion exchange resin with doses of 16 mg·Kg^-1^, 32 mg·Kg^-1^ and 64 mg·Kg^-1^ respectively by oral administration, and the control group was orally administered with 0.5% CMC-Na. At 15, 30, 60, and 90 min after administration, the frequency of autonomic activities of each mouse in 3 min were determined. The frequency of autonomic activities in the experimental groups was compared with that in the control group with t-test. 

*The effect of pH sensitive ion exchange resin on heart rate, blood pressure, ECG and breathing of anesthetic cats*

24 cats were evenly divided into four groups with similar sex and weight, the control group and the low, medium and high dose group respectively. Cats were anesthetized by intraperitoneal injection of 20% urethane (1.0 g·Kg^-1^) and fixed at back. The anterior portion skin of nece was disinfected conventionally, and a central longitudinal incision with length of approximately 4~5 cm was made below the prominentia laryngea. The carotid artery on one side was separated, and an artery intubation was inserted and connected to RM6240 biological signal acquisition and processing system through the pressure transducer. The mean arterial pressure (MBP, mmHg) was recorded. A transverse incision was made at epigastric side with the length of approximately 4~5 cm and the muscle layer were incised to open the abdominal cavity. The position of duodenum was determined for administration. At the same time, ECG electrode was connected to record ECG and heart rate and processus xiphoideus was separated and connected with muscle tension transducer to synchronously record the breathing frequency and breathing depth.

The administration was not conducted until each index became steady. Then 5 mg·Kg^-1^, 10 mg·Kg^-1^, 20 mg·Kg^-1^ of pH sensitive ion exchange resin were injected with 4 mL·Kg^-1^ through duodenum to jejunum. After administration we continuously observed for 90 min to respectively record changes of indexes above of each cat before the administration, 5, 15, 30, 60 and 90 min after the administration. The significance of each index between experimental group and control group was obtained by t-test of paired data. 

## Results and Discussion

*Acute toxicity test by oral administration*

The results showed that the 20 mice in the experimental group exhibited no obvious poisoning representation but normal free-moving, eating and drinking activities. After observed for 2 weeks, the mice were all alive and weighed at the 15th day. Compared with control group, the weight of mice in the experimental group whose dose is 18.84 g/Kg didn’t have significant difference. Their weight are at the range of 21.1 ± 2.2 g, while the group’s are at the range of 20.7 ± 3.5 g (P > 0.05). After the mice were sacrificed, no visible pathological change in major organs was observed. The LD_50 _of pH sensitive ion exchange resin after oral administration was more than 18.84 g·Kg^-1^. At 2 weeks after oral administration, there was no significant difference in the weight of mice between the experimental group and the control group.

*General pharmacological studies*

*The effect of pH sensitive ion exchange resin on nervous system of mice after oral administration*

Average score of each group was calculated, the grading standard of the muscle twitching, salivation and nystagmus in mices defines that none phenomenon scores 0, lightly scores 4, and strong scores 8. The effect of pH sensitive ion exchange resin and the 0.5% CMC-Na (the control group) on nervous system in mice didn’t be observed with the items of Righting reflex, Passive state, Muscle twitching, salivation and nystagmus at the time of 0, 5, 10, 30, 60, 90(min) after oral administration. The results indicated that with the dose used in this experiment by oral administration, pH sensitive ion exchange resin didn’t have significant influence on the nervous system and general behavior of sober animals.

*The effect of pH sensitive ion exchange resin on the functional coordination of mice (rotating rods method)*

The doses of the pH sensitive ion exchange resin are 16 mg·Kg^-1^, 32 mg·Kg^-1^ and 64 mg·Kg^-1^, the control group didn’t give a dose as a comparison. The number is 10 in every level. The result indicated that the pH sensitive ion exchange resin didn’t have significant influence on the coordinated motion (rotating rods method) of mice with the chosen dose range by oral administration.

*The effect of pH sensitive ion exchange resin on hypnosis of mice treated with nembutal at subliminal dose*

The doses of the pH sensitive ion exchange resin are 16 mg·Kg^-1^, 32 mg·Kg^-1^ and 64 mg·Kg^-1^, the control group didn’t give a dose as a comparison. The number is 20 in every level. The result shows that there is one mice falling asleep in every level of two groups, then the rate of falling asleep is 5%. In summary, the result indicated that the pH sensitive ion exchange resin didn’t have obvious hypnotic effect on the mice treated with nembutal at subliminal dose.

*The effect of pH sensitive ion exchange resin on the autonomic activities of tested mice*

The result was shown in Table 1. The result showed that there was no significant difference in the frequency of autonomic activities before and after the administration of pH sensitive ion exchange resin, compared with the control group.

**Table 1:** Effect of pH sensitive ion exchange resin on autonomic activities in mice（$\bar{X}$±SD，n=10）

**Group**	**Dose** **（** **mg/Kg** **）**		**After administration** **（** **min** **）**				
		**0**	**15**	**30**	**60**	**90**
Control	-	126.0±51.9	118.6±55.5	77.3±34.9	61.0±36.9	48.8±37.2
pH sensitive ion exchange resin	16	118.7±54.6	91.0±35.3	66.5±26.1	44.1±18.4	61.1±25.2
	32	119.7±45.9	80.4±35.7	61.2±23.9	40.5±25.4	47.6±20.9
	64	126.9±37.5	107.2±48.6	85.1±47.4	83.9±45.7	35.6±22.6

P>0.05, compared with Control.

*The effect of pH sensitive ion exchange resin on heart rate, blood pressure, ECG and breathing of anesthetic cats*

Results were shown in Table 2-10. Cats have several advantages for the cardiovascular investigation, such as blood pressure is constant, the drug reaction sensitive; Blood vessels and nerves thicker, and the tube wall elasticity, easy to operation and suitable for analysis of the drug to the mechanism of action of cardiovascular system. 

Mice were orally administered with 16 mg·Kg^-1^, 32 mg·Kg^-1^ and 64 mg·Kg^-1^ of pH sensitive ion exchange resin and there was no significant influence on mice nervous system, general behavior, function coordination, hypnotic effect treated with nembutal at subliminal dose and frequency of autonomic activities. Within the 90 min after 5 mg·Kg^-1^, 10 mg·Kg^-1^, 20 mg·Kg^-1^ pH sensitive ion exchange resin was injected to cat duodenum, the heart rate, blood pressure, breathing and ECG of the cats didn’t make significant changes in each experimental group compared with the control group. 

**Table 2:** Effect of pH sensitive ion exchange resin on heart rate in anesthetized cats（$\bar{X}$±SD，vices /min，n=6）.

	**Group and Dose**			
**Time**	**0.5% CMC-Na**	**pH sensitive ion exchange resin**		
	4ml/kg	20mg/kg	10mg/kg	5mg/kg
0′	181±18	198±26	205±14	190±23
5′	180±21	200±34	191±27	187±22
15′	181±23	205±32	197±18	199±24
30′	186±21	208±30	203±15	204±25
60′	206±23	208±31	200±22	207±26
90′	204±17	219±29	199±24	214±40

P>0.05, compared with Control

**Table 3:** Effect of pH sensitive ion exchange resin on mean arterial blood pressure (MAP) in anesthetized cats （$\bar{X}$±SD，Kpa，n=6）.

**Time**	**Group and Dose**			
	**0.5% CMC-Na**	**pH sensitive ion exchange resin**		
	4 mL/Kg	20 mg/Kg	10 mg/Kg	5 mg/Kg
0′	30±3	30±4	29±5	30±4
5′	31±3	29±3	28±4	29±3
15′	30±4	30±3	29±4	28±4
30′	30±3	29±3	30±4	27±4
60′	28±4	28±3	30±4	27±4
90′	28±4	28±3	20±4	27±5

P>0.05, compared with Control

**Table 4:** Effect of pH sensitive ion exchange resin on breathing rate in anesthetized cats （$\bar{X}$±SD，vices/min，n=6）

**Time**	**Group and Dose**			
	**0.5% CMC-Na**	**pH sensitive ion exchange resin**		
	**4 mL/Kg**	**20 mg/Kg**	**10 mg/Kg**	**5 mg/Kg**
0′	52±36	36±20	56±30	51±28
5′	43±16	33±16	42±16	41±13
15′	46±13	35±17	44±13	44±16
30′	50±20	40±21	55±23	47±22
60′	57±24	41±19	48±22	52±22
90′	54±23	42±20	45±11	51±25

P>0.05, compared with Control

**Table 5:** Effect of pH sensitive ion exchange resin on respiratory depth in anesthetized cats（$\bar{X}$±SD，g，n=6）.

**Time**	**Group and Dose**			
	**0.5% CMC-Na**	**pH sensitive ion exchange resin**		
	**4 mL/Kg**	**20 mg/Kg**	**10 mg/Kg**	**5 mg/Kg**
0′	6.66±1.69	7.60±1.76	7.51±1.93	7.17±1.91
5′	6.98±1.61	7.71±2.34	8.43±2.30	7.51±1.95
15′	6.34±2.16	7.98±2.26	9.13±2.49	8.02±2.62
30′	7.60±2.16	8.33±1.26	9.68±2.24	8.36±2.65
60′	7.76±2.01	9.21±1.88	9.91±2.93	9.16±2.24
90′	7.36±2.66	9.35±2.01	8.88±2.99	8.54±2.16

P>0.05, compared with Control

**Table 6:** Effect of pH sensitive ion exchange resin on P wave of electrokardiogram in anesthetized cats（$\bar{X}$±SD, mV，n=6）

**Time**	**Group and Dose**			
	**0.5% CMC-Na**	**pH sensitive ion exchange resin**		
	**4 mL/Kg**	**20 mg/Kg**	**10 mg/Kg**	**5 mg/Kg**
0′	0.16±0.03	0.16±0.05	0.19±0.04	0.19±0.04
5′	0.18±0.04	0.16±0.06	0.20±0.05	0.17±0.04
15′	0.17±0.03	0.18±0.05	0.22±0.05	0.18±0.05
30′	0.17±0.03	0.18±0.05	0.21±0.06	0.19±0.05
60′	0.18±0.07	0.17±0.04	0.22±0.05	0.20±0.04
90′	0.21±0.07	0.17±0.05	0.22±0.07	0.21±0.04

P>0.05, compared with Control

**Table 7:** Effect of pH sensitive ion exchange resin on T wave of electrokardiogram in anesthetized cats （$\bar{X}$±SD mV，n=6）

**Time**	**Group and Dose**			
	**0.5% CMC-Na**	**pH sensitive ion exchange resin**		
	**4 mL/Kg**	**20 mg/Kg**	**10 mg/Kg**	**5 mg/Kg**
0′	0.39±0.14	0.28±0.10	0.30±0.18	0.39±0.24
5′	0.46±0.13	0.34±0.08	0.33±0.20	0.43±0.26
15′	0.52±0.28	0.30±0.08	0.31±0.19	0.41±0.23
30′	0.46±0.23	0.30±0.08	0.31±0.19	0.39±0.23
60′	0.39±0.23	0.29±0.08	0.33±0.23	0.37±0.21
90′	0.42±0.24	0.29±0.08	0.34±0.25	0.39±0.23

P>0.05, compared with Control

**Table 8:** Effect of pH sensitive ion exchange resin on QRS wave of electrokardiogram in anesthetized cats（$\bar{X}$±SD，ms，n=6）.

**Time**	**Group and Dose**			
	**0.5% CMC-Na**	**pH sensitive ion exchange resin**		
	**4 mL/Kg**	**20 mg/Kg**	**10 mg/Kg**	**5 mg/Kg**
0′	18±5	19±5	19±9	22±6
5′	21±5	20±6	19±7	19±5
15′	21±6	21±5	19±5	22±4
30′	19±9	19±5	19±5	19±3
60′	19±5	19±2	18±2	20±4
90′	18±6	17±2	17±5	18±6

P>0.05, compared with Control

**Table 9:** Effect of pH sensitive ion exchange resin on P-R interval of electrokardiogram in anesthetized cats（$\bar{X}$±SD, ms，n=6）.

**Time**	**Group and Dose**			
	**0.5% CMC-Na**	**pH sensitive ion exchange resin**		
	**4 mL/Kg**	**20 mg/Kg**	**10 mg/Kg**	**5 mg/Kg**
0′	53±9	53±5	49±9	46±3
5′	48±9	55±7	48±4	50±7
15′	49±3	53±7	49±5	47±7
30′	50±7	52±3	49±5	48±6
60′	45±6	53±6	48±4	49±6
90′	50±9	48±7	51±4	47±4

P>0.05, compared with Control

**Table 10:** Effect of pH sensitive ion exchange resin on Q-T interval of electrokardiogram in anesthetized cats（$\bar{X}$±SD, ms，n=6）

**Time**	**Group and Dose**			
	**0.5% CMC-Na**	**pH sensitive ion exchange resin**		
	**4 mL/Kg**	**20 mg/Kg**	**10 mg/Kg**	**5 mg/Kg**
0′	251±33	246±47	236±24	255±27
5′	251±29	253±64	247±30	252±26
15′	247±26	241±49	243±29	245±31
30′	241±25	241±40	233±22	240±29
60′	231±26	236±39	248±37	223±21
90′	225±35	217±27	235±30	231±24

P>0.05, compared with Control

## Conclusion

In the present study, *in-vivo *pharmacological and toxicological researches were investigated for the novel pH sensitive ion exchange resin. The acute toxicity study results showed that the LD_50_ of pH sensitive ion exchange resin after oral administration was more than 18.84 g·Kg^-1^, the general pharmacological studies showed that pH sensitive ion exchange resin had no influence on the nervous system of mice, the functional coordination of mice, the hypnosis of mice treated with nembutal at subliminal dose, the autonomic activities of tested mice, and the heart rate, blood pressure, ECG and breathing of the anesthetic cats. The desirable pharmacological and toxicological behaviors the pH sensitive ion exchange resin exhibited indicate that this novel formulation has great biocompatibility and is possible for clinical use. 
